# Virtual Reality as a Possible Tool for the Assessment of Self-Awareness

**DOI:** 10.3389/fnbeh.2019.00062

**Published:** 2019-04-04

**Authors:** Manuel Muratore, Cosimo Tuena, Elisa Pedroli, Pietro Cipresso, Giuseppe Riva

**Affiliations:** ^1^Applied Technology for Neuro-Psychology Laboratory, Istituto Auxologico Italiano, Milan, Italy; ^2^Department of Psychology, Università Cattolica del Sacro Cuore, Milan, Italy

**Keywords:** self-awareness, virtual reality, assessment, neurodegenerative disorders, anosognosia

## Abstract

The absence of self-awareness is a crucial aspect in the symptomatology of various neurodegenerative disorders. This characteristic becomes relevant due to the strong implications it has on the patient’s quality of life, on the effects that functional dependence has on the caregiver and on the efficacy of the therapy. Faced with a construct as complex as self-awareness, there are in the literature investigations on different aspects of this phenomenon, such as the creation of cognitive models, the study of the neural substrate and the research of appropriate assessment methods that can reliably detect this function. With regard to the assessment methods, there are methodologies in the literature that provide complementary information. The first modality is a quantitatively online measurement based on the discrepancy between the estimate of the patient of his performance and his actual performance, but often neglecting the ecological validity and the real functioning of the subject. The second kind collecting subjective information on the actual daily functioning of the patient resulting from clinical observation or interviews with the subject and caregivers, but obtaining offline information on the functioning of the subject, liable to bias that may imply an overestimation or underestimation of subject’s ability. The absence of acknowledged metacognitive functional assessment with normative data to evaluate awareness winks at the emerging and increasingly consistent use of virtual reality (VR) also in the context of cognitive research and clinical assessment. This article aims to make a theoretical proposal regarding the use of this innovative and promising tool as a supplement to the assessment methods of self-awareness.

## Self-Awareness in Neurodegenerative Disorders

Self-awareness is defined by Morin (2011) as the ability of the individual to bring attention to a series of aspects concerning themselves, such as his own behavior, emotions, personality traits, cognitive abilities, goals, perceptions, bodily sensations. In the context of neurodegenerative diseases, it is addressed as awareness of one’s own illness, of one’s own deficits, both cognitive, physical, behavioral or concerning the emotional sphere (Rosen, 2011). This phenomenon, called anosognosia, is very common in neurodegenerative diseases such as Alzheimer’s disease (AD) and the behavioral variant of frontotemporal dementia (bvFTD). In patients with AD, anosognosia concerns, in particular, the absence of awareness of difficulties on the activities of daily living (ADL) and impaired cognitive functions, such as episodic memory and spatial cognition (Starkstein et al., 2006). Consequently, the research focused on the analysis of the awareness of one’s own memory capacity, also called meta-memory (Zamboni and Wilcock, 2011; Cosentino et al., 2015). In bvFTD, wherein the self-awareness deficit emerges in very early stages and appears to have even more important entity than in AD, awareness of changes in behavioral, emotional and social skills is often assessed (Neary et al., 1998; Desmarais et al., 2018).

Lack of awareness has very important implications on several aspects of the management of the disease, for example, the exposure of the patient to risky situations due to a wrong evaluation of their abilities, less adherence and collaboration to treatment, difficulty in exploiting the proposed strategies which often results in reduced functional autonomy and a consequent greater burden on the caregiver (Asmus et al., 2006). Moreover, this is an aspect that often precedes the cognitive and functional decline. Therefore, many researches have investigated even those condition with a high probability of conversion in neurodegenerative disorders, such as MCI, in which it is present, rather than an evident anosognosia, a reduced self-awareness of one’s cognitive abilities, an element that is associated with an increase in the probability of development of dementia (Tabert et al., 2007). Before that a significant functional and cognitive decline occurs in these patients, an accurate assessment of this condition would allow formulating treatments useful to the intervention (Cummings et al., 2013; Jessen et al., 2014).

### The Traditional Assessment of Self-Awareness

In parallel with the current lack of clarity about the psychological mechanisms and the neural networks that underlie the processes of self-awareness, there is no acknowledged method of measurement that allows quantifying this construct (Rosen, 2011; Sunderaraman and Cosentino, 2017). To date, there are three main evaluation methods in the literature, which are complementary to each other due to the strengths and weaknesses of the type of information that can be obtained. The first modality, related to clinical studies, involves the evaluation of a clinician through using structured or unstructured interviews (Prigatano, 2010); the second one provides a discrepancy measure between the self-report of the subject and that of a caregiver through questionnaires that investigate an aspect of functioning of the patient (Hart et al., 2003; Orfei et al., 2010; Zamboni et al., 2012). Through these first two modes, we obtain information from subjective evaluations, which are therefore subject to bias due to a series of factors (e.g., caregiver burden, limited interaction with the patient, errors in reporting information, etc.) that could lead to overestimation or underestimation of the patient’s abilities. Furthermore, this information refers to the functioning of self-awareness in general, not related to a specific task (in an offline fashion), although they refer to the functioning of the subject in his natural environment, theoretically with greater ecological validity. The third modality, mainly used in experimental studies dealing with meta-cognition, is based on the quantification of the accuracy of the subject in judging previously or after his performance to a standardized neuropsychological task (typically memory or executive functions) and comparing it with its actual performance (Fragkiadaki et al., 2016). This methodology has the undoubted advantage of being able to have an online quantification of the subject’s self-awareness, but at the same time, the used tasks and the traditional neuropsychological tests are useful for evaluating specific cognitive domains and deficient in an ecological evaluation of functional abilities. Furthermore, neuropsychological testing is lacking in the assessment of social skills, which are often affected by anosognosia, especially in bvFTD, in which these deficits are among the first to appear (Eslinger et al., 2005). Despite this, according to the study conducted by Levy et al. (2018), the methodology based on evaluation by a clinician is more associated with brain areas and with executive dysfunction evaluations, both theoretically associated with self-awareness. Furthermore, it is more associated with neuropsychological test performance than the discrepancy method used with the Frontal Systems Behavioral Scale (Levy et al., 2018). In the next section, the properties and technical features of VR will be described and proposed as a method for assessing self-awareness that could compensate for the limitations of the traditional ways above described, integrating their points of strength.

## Self-Awareness Assessment: The Potential of Virtual Reality

Rizzo et al. (1998) has defined virtual reality (VR) as an advanced computer interface that allows the user to interact and become immersed within computer-generated simulated environments.

The VR label contains multiple technological solutions with different characteristics that can comply with specific research and clinical practice requirements (Li et al., 2017). Devices types can be categorized according to the level of immersion they can induce. Non-immersive systems present the virtual environment through a desktop, they are the simplest and relatively cheaper systems available. Immersive systems use devices, such as head-mounted displays (HMDs), that visually isolating user providing a more complete experience, allowing to perceive a 3D stereoscopic images, to detect position in the virtual environment *via* motion tracking sensors integrated into the helmet and providing different levels of interaction with the environment (Table 1). Semi-immersive systems, such as the Cave Automatic Virtual Environment (CAVE), a system that use projectors providing a stereo image of a 3D scene directed on three or more walls of a room, cost more than the other systems, but give a higher sense of reality thanks to the illusion of technological non-mediation (Cipresso et al., 2018). The different kinds of HMDs can require from the simple use of a smartphone to the more powerful systems that need to be connected to a PC. Interaction modes also vary between device types, allowing from simple exposure to an environment up to the manipulation of items in the environment through one or two controllers. The more complex systems allow the planning of more interactive tasks but, besides having a higher cost, implies a greater difficulty in the development of the program. However, there are platforms for the development of VR environments, such as Neuro VR (Riva et al., 2007), suitable for use by non-experts, with the possibility of creating high-quality environments.

**Table 1:** Comparison of reality systems.

	PC-based			Mobile-based			Console-based	Standalone mobility		
Mobilty required										
System	Oculus Rift	HTC Vive/Vive Pro	Microsoft Mixed Reality	Samsung Gear VR	Google Cardboard	Google Daydream	Playstation VR	Oculus Go	Oculus Quest	Mirage Solo
Cost	399 US$	499/799 US$	249/449 US$	99 US$	10–50 US$	69–149 US$	299 US$	199 US$	399 US$	299 US$
Hardware requirements	High End PC (>1,000 US$)	High End PC (>1,000 US$)	Mid Level PC (>600 US$)	High End Samsung Phone (>600 US$)	Middle/High end Android phone or iPhone (>299 US$)	High End Android Phone (>499 US$)	PS4 (299 US$) or PS4 Pro (399 US$)	None (Internal Snapdragon 821 processor)	None (Internal Snapdragon 835 processor)	None (Internal Snapdragon 835 processor)
Resolution	2,160 × 1,200	2,160 × 1,200/2,880 × 1,660	2,880 × 1,440	2,560 × 1,440	Depends from the phone (minimum 1,024 × 768)	Depends from the phone (minimum 1,920 × 1,080)	1,920 × 1,080	2,560 × 1,440	2,560 × 1,440	2,560 × 1,440
Refresh rate	90 Hz	90 Hz	90 Hz	60 Hz	60 Hz	90 Hz minimum	120 Hz	72 Hz	72 Hz	75 Hz
Field of view	110°	110°	100/110°	101°	from 70°	96°	100°	90°	100°	100°
Body tracking	Medium/High: head tracking (rotation) and positional tracking (forward/backward)	High: head tracking (rotation) and volumetric tracking (full room size– 15 ft x 15 ft—movement)	Medium/High: head tracking (rotation) and positional tracking (forward/backward)	Medium: head tracking (rotation)	Medium: head tracking (rotation)	Medium: head tracking (rotation)	Medium/High: head tracking (rotation) and positional tracking (forward/backward)	Medium: head tracking (rotation)	Medium/High: head tracking (rotation) and positional tracking (forward/backward)	Medium/High: head tracking (rotation) and positional tracking (forward/backward)
User interaction with VR	High (using a joystick or controllers)	High (using controllers)	High (using a joystick or controllers)	Medium (using gaze, a built in pad or joystick)	Low (using gaze or a button)	Medium (using gaze or joystick)	High (using a joystick or controllers)	Medium (using gaze, a built in pad or joystick)	High (using a joystick or controllers)	Medium (using gaze, a built in pad or joystick)
Software availability	Oculus Store	Steam Store	Microsoft Store	Oculus Store	Google Play or IOS Store	Google Play	Playstation Store	Oculus Store	Oculus Store	Google Play

This technological tool is proposed as a mean that can improve different aspects of traditional neuropsychological assessment (Riva, 1997, 2002). Traditional neuropsychological tests investigate isolated cognitive functions and often under artificial conditions that imply low ecological validity and consequently provide poor information on the actual daily functioning of the subject. The VR, on the other hand, allows using a series of settings that simulate those of the real world, such as cities, supermarkets, workstations and domestic environments, for the assessment of complex capabilities and the actual functioning of the patient. This new technologic tool allows the use of interactive, multimodal sensory stimuli with a high degree of ecological validity and provides a high degree of control over the content variables and stimulus delivery and responses measurement and storage in clinical assessment or rehabilitation settings (Lee et al., 2003; Rizzo et al., 2004; Bohil et al., 2011). According to the study of Lopez Maite et al. (2016), the functional assessment with VR provides a mean to objectively experimentally evaluate the functional impact of the disorders in situations close to the constraints of real life, but offers the advantage to avoid the dangers of real-life environments, becoming a suitable tool for testing vulnerable adults, such as patients with neurodegenerative disease (Elkind et al., 2001). Another important feature of this technology is the potential impact of the VR experience. There is evidence of a good learning of the skills trained in VR and that these skills can be transferred to similar tasks in the real world (McComas and Sveistrup, 2002), making it an interesting tool both for cognitive assessment as for rehabilitation.

There are several studies that have used VR in the evaluation of cognitive functioning, both in healthy subjects and patients with brain injury or with neurodegenerative diseases, of the latter, patients with AD, bvFTD and Parkinson’s disease. Moreover, according to a study by Flynn et al. (2003), aimed at assessing whether the use of VR as a clinical tool is applicable to people with dementia, it seems that this kind of patients experience a good sense of presence, but above all that VR does not involve problems with physical or psychological well-being for these kinds of patients. The feasibility of VR has been studied to extend it also to the application on non-AD dementias, less considered in the adoption of this technology. Mendez et al. (2015), in a study evaluating the feasibility of a virtual environment through HMD, state that the characteristics and procedures of VR are feasible and well tolerated by their sample of patients with bvFTD, who also reach sufficient levels of presence.

Cognitive assessment studies with VR focused on episodic memory aspects (Plancher et al., 2012; Plancher and Piolino, 2017), spatial navigation skills (Cushman and Duffy, 2008; Serino et al., 2015), executive functions (Davison et al., 2018), up to the evaluation of skills in daily life activities (Flynn et al., 2003; Lee et al., 2003; Allain et al., 2014). Interestingly, Allain et al. (2014) suggest that by its peculiarities VR testing can point out subtle deficits, often not detected using traditional neuropsychological tests (Pallavicini et al., 2015), that allows a more accurate evaluation and to plan a more targeted rehabilitation.

VR and its benefits have been successfully combined and integrated with different methodologies of assessment and rehabilitation of several cognitive functions. Unfortunately, to date, there are no VR systems that directly assess the self-awareness deficit. There are, however, some studies that provide insights for the use of VR for this purpose. Lloréns et al. (2013) have developed a virtual board game with a multi-touch table for the rehabilitation of self-awareness in patients with acquired brain injury. In this competitive game, two groups of patients will have to answer questions (e.g., about the implications of brain damage or the limits it entails) to reach first the end of a goose game-like path. Their methodology uses the virtual tool to increase involvement, participation and interaction among multiple patients to strengthen the pedagogical process and recognition of the limits on which their treatment approach is based (Lloréns et al., 2013). Another example is the study by Mendez et al. (2015), in which a comparison is made between a traditional insight assessment and an insight assessment through a VR interview in patients with bvFTD. The patient is immersed through an HMD in a virtual environment where there are five avatars and has to answer a series of avatar questions, including those related to the insight assessment of the UCLA Structured Insight Interview. The results show that subjects provide longer, more elaborate answers when questions are expressed by avatars than in the real world, providing more information and demonstrating greater self-awareness in the virtual condition (Mendez et al., 2015). Although both of these approaches suggest the usefulness of using VR in the investigation and treatment of self-awareness, the virtual tool is used for an intervention that is not performance-centered. Through the virtual game in the Lloréns study, awareness of deficits is raised through a psychoeducational process rather than exploiting VR to allow a more experiential process based on comparing one’s expectations of functioning with one’s actual performance. The virtual assessment in Mendez’s study is also done by collecting information offline through a virtual interview and not from the patient’s assessment during the performance.

The overcoming of this aspect emerges in an interesting study by Koenig et al. (2011) who use a VR task for the assessment or training of short-term memory and the ability to imagine different perspectives in 3D space Virtual Memory Task (VMT). The object of the authors was to create a clinical tool designed to have a higher ecological validity than traditional tests, to be able to keep higher motivation for patients to practice the task frequently in a meaningful test environment and record precise measurements in 3D space for analyzing the task’s results. The task consists in a coding phase, wherein subject needs to memorize the exact position of some target object on a table, and a test phase, wherein the target objects position and point of view was changed, and the participant should precisely move the items back to the initial locations. An important aspect of this protocol is that was recorded a distance error score for each trial (calculated for each target by finding the distance between the participant’s answer and the object’s original position during the coding phase) and that a feedback was given to the patient after each session. Although self-awareness issues are not part of the focus of this study, the authors report interesting information about the induced effect of using their task. In fact, in addition to the results concerning the validation of the task, the authors highlight how the execution of this exercise led to a significant change in the awareness of the cognitive deficit in several participants. This result is attributed by the authors to the highly realistic semi-familiar virtual environment, that allows making comparisons to the real environment whenever participants were skeptical about test results or the nature of the task. It is also plausible that the use of viewable and quantifiable feedback has also played a role in this process. This study, compared to the previous one, carries out “unintentionally” a method of intervention on self-awareness based on the use of VR as an environment in which to perform an ecological performance. Thanks to the high ecological characteristics of the proposed condition and to the possibility of recording and displaying the performance outputs graphically, this simple task is able to stimulate objective self-evaluation through comparison and therefore the awareness of the patient’s deficit.

Despite the absence of studies that directly test VR systems for self-awareness assessment, taken together these studies provide interesting premises for implementing the assessment procedure with this type of technology.

**Figure 1:**
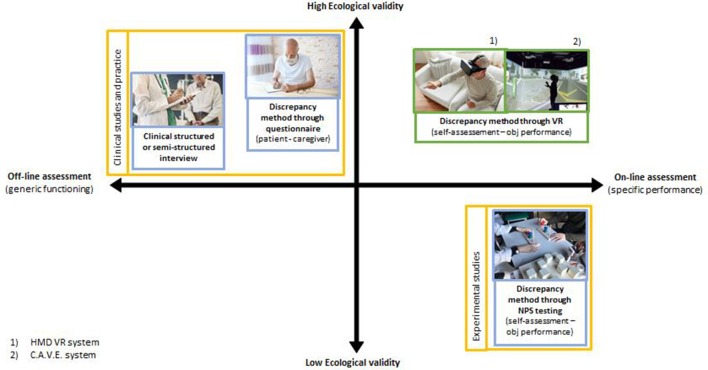
Comparison of self-awareness assessment method.

Thanks to its features, a self-awareness assessment method supported by a VR system would allow to overcome the limits and integrate the positive sides of traditional assessment methodologies. In particular, as already introduced above, both the assessment by a clinician through interview, and the method based on the measure of discrepancy between the answers to questionnaires by the patient and the caregiver, have the advantage of providing information on the functioning of the subject in real daily situations (having high ecological validity), but both methods rely on offline subjective evaluations, regarding general cognitive functioning rather than specific performance and liable to judgment bias that can lead to a wrong assessment. On the contrary, a VR system would allow to objectively record and quantify the subject’s performance, to make a comparison and to measure the discrepancy between the subject’s evaluation and his effective performance. Instead, the traditional method of measuring the discrepancy between subjective estimation and actual patient performance in traditional neuropsychological assessment tests has the advantage of being able to carry out an online and quantifiable assessment of performance, but it uses extremely specific material for specific cognitive domains and is often lacking in functional ecological assessment. In this sense, the VR system would compensate low ecological validity of traditional tests, thanks to controllable environments that simulate realistic conditions, recognizable as concrete by the subject, which therefore leads to a quantifiable ecological assessment of the awareness of cognitive deficits and possible repercussions in realistic everyday situations (Figure 1).

## Conclusion

Carrying out an evaluation method based on the comparison between the subject’s prediction of his performance and his actual performance in high-ecological VR tasks would provide an effective method for reliable self-awareness detection. Moreover, the presence of cognitive tests in VR for the evaluation of different cognitive functions (e.g., episodic memory, spatial cognition, executive functions), would allow to assess the degree of awareness for the compromised functions associated with the patient’s pathology (Zhang et al., 2001; Parsons et al., 2008). This combination through the use of VR would provide the advantages of traditional online evaluation, i.e., the possibility of having quantitative measures of performance, and those of an evaluation related to a functioning comparable to the actual daily one of the subject such as the one emerging from the method based on discrepancy between the information of the subject and those of the caregiver. This approach is encouraged by the affordable prices of several VR devices and available open source software that allows non-expert users to modify pre-existing virtual environment with respect to the needs of clinical or research settings (Riva et al., 2007). Furthermore, the specific features of the different systems, such as the possibility of moving in the real environment, the detection of body movements, levels and modes of interaction with the virtual environment, help to different requests (simple exposure, spatial navigation of the environment, need to interact with elements of the environment) related to the study or clinical assessment of the various cognitive functions.

Regarding the frequency with which the lack of self-awareness occurs in degenerative disorders, and the implications it has on patient management and the planning of an effective therapeutic process (Piras et al., 2016), we believe that VR can be a very interesting means to bring the assessment of this capacity at a higher level of clinical and research reliability and utility.

## Author Contributions

MM wrote the first version of the manuscript. CT, EP and PC revised and critically contributed to the article. GR supervised and revised the last version of the article.

## Conflict of Interest Statement

The authors declare that the research was conducted in the absence of any commercial or financial relationships that could be construed as a potential conflict of interest.
